# Functional outcomes in children related to self-care, mobility, and social function after stroke in early childhood: a cohort study

**DOI:** 10.1590/0004-282X-ANP-2021-0019

**Published:** 2022-01-31

**Authors:** Larissa Audi Teixeira Mota, Daniela Rodrigues Baleroni Silva, Luzia Iara Pfeifer

**Affiliations:** 1 Universidade de São Paulo, Faculdade de Medicina de Ribeirão Preto, Departamento de Neurociências e Ciências do Comportamento, Ribeirão Preto SP, Brazil. Universidade de São Paulo Faculdade de Medicina de Ribeirão Preto Departamento de Neurociências e Ciências do Comportamento Ribeirão Preto SP Brazil; 2 Integra KIDS Terapias Avançadas em Reabilitação Infantil, Ribeirão Preto SP, Brazil. Integra KIDS Terapias Avançadas em Reabilitação Infantil Ribeirão Preto SP Brazil

**Keywords:** Stroke, Child, Self-Care, Locomotion, Social Skills, Acidente Vascular Cerebral, Criança, Autocuidado, Locomoção, Habilidades Sociais

## Abstract

**Background:**

Stroke has been increasingly recognized as an important morbidity and mortality factor in neonates and children. Children have different and more diverse risk factors than adults, commonly related to an underlying disease. Stroke may compromise functional capacity in children. Few studies have focused on functional outcomes related to activities and participation.

**Objectives:**

To investigate post-stroke functionality of children related to self-care, mobility, and social function.

**Methods:**

We assessed the functional outcome of 14 children younger than 7.5 years who suffered a stroke in early childhood through the use of the Pediatric Evaluation of Disability Inventory (PEDI).

**Results:**

The average age of the sample at assessment was 3.6 ± 1.4 years (2 - 6 years). The average scores in the PEDI functional domains of self-care, mobility, and social function were, respectively, 37.6 ± 15.4, 36.2 ± 15.4, and 48.7 ± 11.1. Children showed age-appropriate functional outcomes in the PEDI functional domains: 71.4% of them in self-care and mobility and 92.9% in social function. Children with bilateral injuries (p = 0.05) and longer hospital stays (r = -0.79, p = 0.001) showed the worst scores in ​​PEDI's social function domains.

**Conclusions:**

Overall, our sample of preschool children showed age-appropriate functional outcomes on self-care, mobility, and social function domains after stroke. However, children with bilateral injuries and longer hospital stays showed the worst scores in social function domains. We recommend focusing on functional rehabilitation to promote activities and participation and to monitor the development of children's social skills after stroke.

## RESUMO

**Antecedentes:**

O acidente vascular cerebral (AVC) tem sido reconhecido como um importante fator de morbimortalidade em neonatos e crianças. As crianças têm fatores de risco diferentes e mais variados que os adultos, comumente relacionados a uma doença subjacente. A funcionalidade das crianças pode estar comprometida após um AVC. Poucos estudos focaram em desfechos funcionais relacionados à atividade e participação.

**Objetivos:**

Investigar a funcionalidade de crianças com AVC, relacionada à autocuidado, mobilidade e função social.

**Métodos:**

Avaliamos a evolução funcional de 14 crianças com idade menor que 7,5 anos com AVC na primeira infância pela aplicação do PEDI.

**Resultados:**

A idade média de nossa amostra na avaliação foi de 3,6 ± 1,4 anos (2 - 6 anos). O escore médio nos domínios de autocuidado, mobilidade e função social do PEDI foram, respectivamente, 37,6 ± 15,4, 36,2 ± 15,4 e 48,7 ± 11,1. As crianças apresentaram evolução adequada para a idade nos domínios do PEDI: 71,4% delas em autocuidado e mobilidade e 92,9% em função social. Piores escores no domínio função social se relacionaram com lesões bilaterais (p = 0,05) e maior tempo de internação (r = -0,79; p = 0,001).

**Conclusões:**

Nossa amostra de crianças em idade pré-escolar mostrou, em geral, evolução funcional adequada para a faixa etária nos domínios de autocuidado, mobilidade e função social. Porém, lesões bilaterais e internações hospitalares mais longas se relacionaram com piores performances no domínio função social. Sugerimos focar na reabilitação funcional e acompanhar o desenvolvimento das habilidades sociais de crianças pós-AVC.

## INTRODUCTION

 Stroke, once considered a health problem in adults, is increasingly recognized as an important morbidity and mortality factor in neonates and children^1^. The annual incidence of pediatric strokes (ischemic and hemorrhagic), considering the neonatal period and childhood, varies from 3 to 25 per 100,000 children in developed countries^1^. The incidence is higher in neonates: 1 in 4,000 live births^1^. While the predominant risk factors in adults include hypertension, diet, diabetes mellitus, obesity, and smoking, among others^2^, children have different and more varied risk factors. Risk factors for childhood stroke (CS) include vasculopathies (such as sickle cell anemia, Moyamoya syndrome and autoimmune disorders), prothrombotic conditions (such as polycythemia, antiphospholipid antibody), heart disorders, genetic and metabolic disorders (such as homocystinuria, Fabry disease), infections, vascular abnormalities, coagulation disorders and tumors^3^.

 Stroke may compromise children's functioning. According to the International Classification of Functioning, Disability and Health (ICF), the concept of functioning includes all body functions and those related to activity and participation^4^. After stroke, the following body functions may be compromised: mental function (attention, information processing)^5^, working memory, visuomotor processing speed^6^, intellectual function^7^, and neuromusculoskeletal/movement-related functions (such as muscle tone)^8^. Activities and participation functions that may be altered include personal care^9^^,^^10^, learning and knowledge application (school problems)^10^^-^^12^, interpersonal relationships (behavioral)^11^, and mobility^13^. After stroke, the child's functionality may be influenced by contextual factors (personal and environmental) such as the child's age and age at stroke, parental education, socioeconomic conditions, family support network, and rehabilitation^8^. 

 Most previous studies have focused on limitations in body functions and structures after a childhood stroke. Few studies have focused on activities and participation functional outcomes and on the influence of the contextual factors on the child's functioning^8^. The few available data (mentioned above) are from international studies^9^^-^^13^, which may not reflect the reality of functional outcome after childhood stroke in developing countries due to socioeconomic and cultural differences. To the best of our knowledge, there are only four studies on functional outcomes after childhood stroke in Brazil. They reported impairments in motor skills, writing, reading, memory^14^^,^^15^, and language^16^; one study on quality of life reported decreased functional capacity^17^. We found no Brazilian studies on functional outcomes in activities of daily living (ADLs) and participation after childhood stroke. There is a need to know the functional outcome of children after stroke to provide the most appropriate intervention focusing on activity and participation skills rather than just improving impairments in body structure/function levels. Thus, the objective of this study was to investigate the functionality of children after a stroke in terms of social function, mobility, and self-care skills.

## METHODS

This was a retrospective longitudinal observational cohort study. We used the STROBE checklist (https://www.strobe-statement.org).

### Participants

The inclusion criteria were: stroke diagnosis (ischemic and hemorrhagic) and age between 6 months and 7.5 years* (*age covered by the evaluation instrument used in the study (PEDI) for the normative score). The exclusion criteria were: no signed informed consent, presence of traumatic brain injury or diffuse brain injury, peri-intraventricular hemorrhage, other causes of cerebral ischemia, associated pathologies with a significant neuropsychomotor development delay such as Down syndrome, West syndrome, and others.

### Instruments

Pediatric evaluation of disability inventory (PEDI)^18^^,^^19^

The PEDI is administered as a structured interview with one of the child's parents/guardian and informs about the children's profile in three functional domains: self-care, mobility, and social function. The PEDI's functional profile consists of three parts; in this study, we applied part I, referring to the child's functional skills. We used the raw score to calculate the continuous score and normative score according to each child's age. The normative score reflects a child's performance concerning a reference sample; it must be between 30 and 70 to be considered age-appropriate. The item maps show the functions of which the child is capable or incapable. The items are arranged on the map in ascending level of difficulty, being the most complex closest to 100. According to the child's raw score and age, the continuous score and standard deviation are plotted on the map. The items to the left of this range are less complex, so we expected the child to be able to perform them.

Brazil economic classification criterion^20^

The Brazil Economic Classification Criterion (BECC) is an economic segmentation instrument. This Criterion differentiates the population in economic classification strata (A1, A2, B1, B2, C1, C2, D, and E). The A1 stratum refers to the best financial condition and the E stratum, the worst economic situation. The classification is based on the family provider's educational level and household characteristics (presence and quantity of some household comfort items).

### Data collection procedures

The Research Ethics Committee of the Ribeirao Preto Medical School of the University of São Paulo approved this study. All participants' parents/guardians were informed about the study and provided a signed informed consent form.

We performed a review of medical data of all childhood stroke cases admitted to the Clinical Hospital of Ribeirao Preto Medical School of the University of São Paulo between 2005 and 2012. This is a tertiary-level university hospital. Then, we reviewed the medical records of selected children to identify childhood strokes. We scheduled the children's assessment for the exact date they would return for a clinical follow-up appointment. Based on the family's preference, children that were not scheduled to return by April 2013 were assessed at home.

We applied the PEDI's part 1 questionnaire in an in-person structured interview with the children's parents/guardians.

### Data analysis

We analyzed the correlation between the PEDI's normative scores in self-care, mobility and social function domains with the following categorical variables: age at stroke (≤1 /> 1 year), injury site (lobar/infratentorial/deep), presence of hydrocephalus (yes/no), presence of intraventricular hemorrhage (yes/no), sex (female/male), stroke type (ischemic/hemorrhagic), affected hemispheres (unilateral/bilateral), and socioeconomic status (Brazil Economic Classification Criterion) using the Mann-Whitney non-parametric test. We also correlated such categorical variables with the child's current age and hospital stay (numerical variables) using the Spearman correlation coefficient. 

## RESULTS

**Figure 1.**
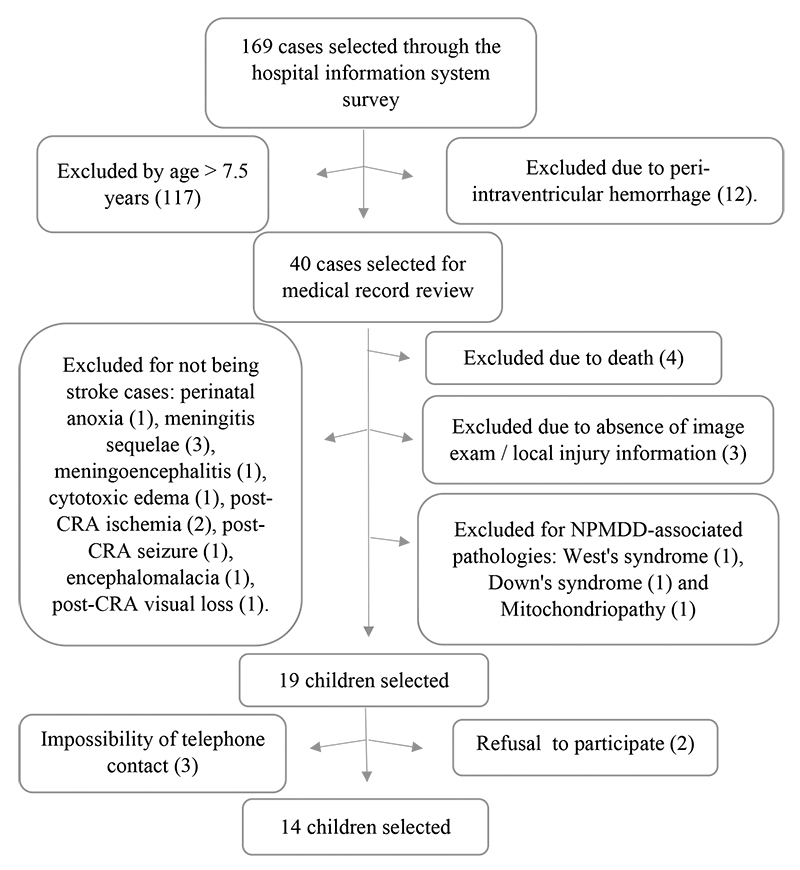
Flowchart for the selection of childhood stroke participants.

CRA: cardiorespiratory arrest; NPMDD: neuropsychomotor development delay.

Figure 1 describes the participants’ screening and selection process.

The mean age at stroke was 1.5 ± 1.4 years (range: 2 days to 4.4 years). The average age at assessment was 3.6 ± 1.4 years (range: 2.2 to 6.3 years). Table 1 describes the participants’ characteristics**.**

**Table 1.** Participants’ characteristics (n=14).

Patients characteristics		Number of patients (n)
Age at stroke	< 1 year	6
	> 1 year	8
Sex	Female	8
	Male	6
Lesion location	Deep	5
	Lobar/Infratentorial	9
Stroke Type	Ischemic	8
	Hemorrhagic	6
Side	Unilateral	10
	Bilateral	4
Intraventricular Hemorrhage	No	12
	Yes	2
Hydrocephalus	No	12
	Yes	2
Socioeconomic status	B1	1
	B2	1
	C	12
School	Yes	9
	No	5
Brothers/sisters	0	4
	1	8
	2	2

We applied the PEDI after an average interval of 2.5 ± 1.3 years post-stroke (range: 1 to 5.5 years). Of 14 children, 7 had another comorbidity in addition to the underlying disease: 6 (42.9%) had epilepsy and 1 (7.1%) had strabismus. Only 4 (28.6%) children underwent physical therapy rehabilitation (one of them had only 10 sessions before discharge). Of these, 2 (14.3%) also received occupational therapy and 1 (7.1%) received speech therapy. At the time of assessment, no child was receiving any type of rehabilitation intervention. 

The average normative score for PEDI's functional skills in the self-care domain was 37.6 ± 15.4; in the mobility domain, it was 36.2 ± 15.4, and in the social function domain, it was 48.7 ± 11.1. Most children scored between 30 and 70 on the normative score profile. Only 4 (28.6%) children did not perform adequately in the self-care domain, 4 (28.6%) in mobility, and only 1 (7.1%) in social function. Among children with age-appropriate functional outcome, the average of normative scores for PEDI's functional skills in self-care, mobility and social function were, respectively, 46 ± 6.8, 43.7± 9.5, and 50.7 ± 8.7. Table 2 presents the children's normative score in self-care, mobility and social function skills. 

**Table 2.** Participants’ characteristics and normative scores in the three PEDI functional domains.

P (n=14)	Age at stroke	Age at assessment (years)	Stroke type	Lesion location	SCFS score	MOFS score	SFFS score
P1	2 d	5	H	Lobar	42.2	29.3*	54.3
P2	1.4 m	2	H	Deep	42.4	39.5	55.6
P3	2.0 m	2	I	Lobar	34.6	41	63.3
P4	4.9 m	4	I	Deep/Infratentorial	21.2*	31	46.6
P5	6.8 m	2	I	Lobar	41.6	36.4	53.1
P6	11.2 m	3	I	Lobar	44.9	43.8	45.2
P7	12.9 m	2	H	Lobar	59.1	57.1	43.1
P8	13.2 m	5	I	Deep	23.3*	<10*	67.3
P9	18.6 m	3	I	Deep	47.3	39.4	45.2
P10	21.1 m	3	I	Lobar/Deep/Infratent.	12.1*	<10*	23.3*
P11	24.3 m	3	I	Infratentorial	46.8	36.2	42.5
P12	38.8 m	6	H	Infratentorial	54.2	56.3	58.3
P13	46.9 m	6	H	Lobar	47.2	56.2	45.3
P14	52.8 m	5	H	Lobar	<10*	22.6*	38.9

P: participant; SCFS: Self-Care Functional Skills; MOFS: Mobility Functional Skills; SFFS: Social Function Functional Skills; I: Ischemic; H: Hemorrhagic; d: days; m: months; *score below that expected for the age (between 30-70).

**Figure 2.**
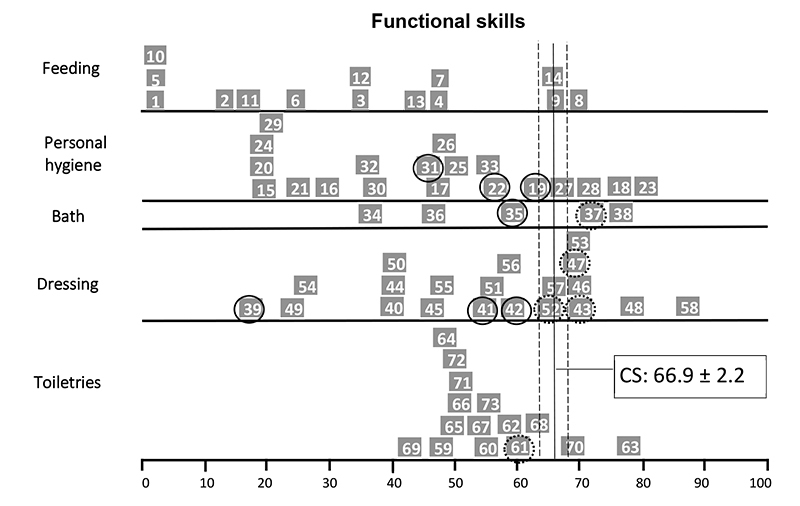
PEDI Item Maps in Self-Care Functional Skills of child P4.

**Figure 3.**
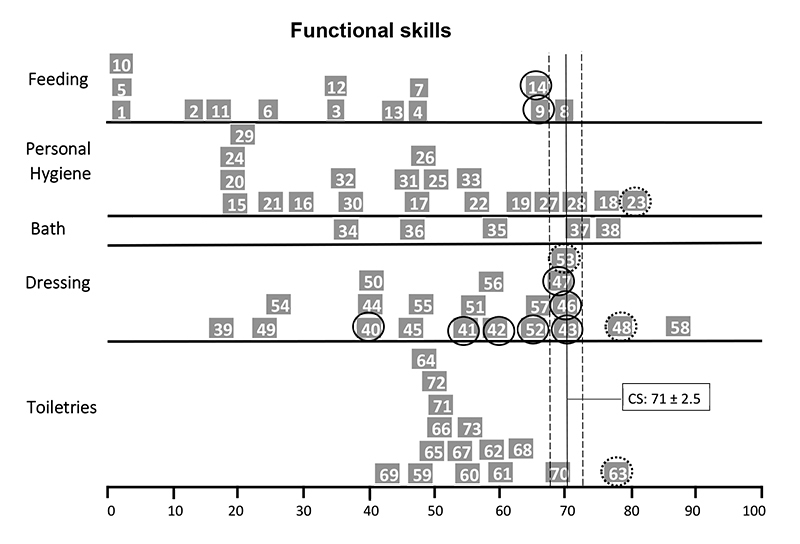
PEDI Items Map in Self-Care Functional Skills of the child P8.

**Figure 4.**
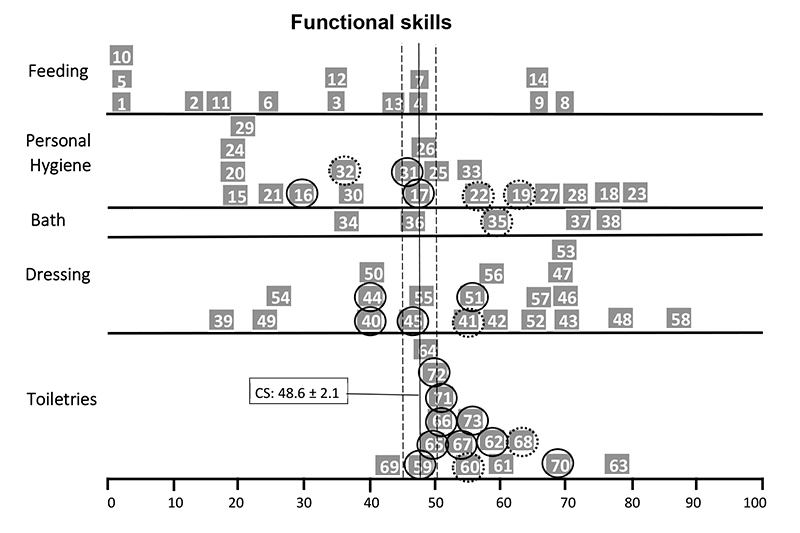
PEDI Items Map in Self-Care Functional Skills of the child P10.

**Figure 5.**
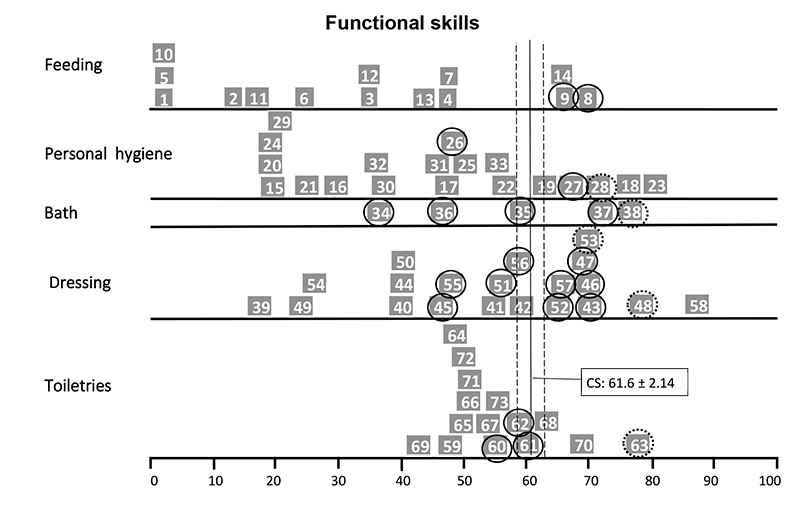
PEDI Items Map in Self-Care Functional Skills of the child P14.

The self-care functional skills item maps of child P4 (Figure 2) showed that she performed below average compared to the normative sample on tasks related to personal hygiene (3 of 20 items), bathing (1 of 5 items) and dressing (3 of 21 items), especially on bimanual tasks or tasks that required movements of the upper limbs up to shoulder level. Similarly, the item map of child P8 showed inadequate performance in feeding (2 of 15 items) and dressing (7 of 21), especially bimanual task items (Figure 3). Child P10’s item map (Figure 4) showed an age-inappropriate performance on personal hygiene (3 items out of 20), dressing (4 of 21 items), and toiletries (9 of 15 items). This child's tasks limitations were related to the neuromotor and cognitive demands of the task's items. Child P14 showed inadequate performance on some feeding (2 items out of 15), personal hygiene (2 of 20 items), and toiletries items (3 of 15 items) and on most bathing (4 of 5 items) and dressing tasks (9 of 21 items), mainly related to routine care with the hemodialysis catheter (Figure 5).

◯ >90% of the children in the age group can do the task; ◌ >75% of the children in the s age group already can do the task; CS: Continue Score; PEDI: Pediatric Evaluation of Disability Inventory.

◯ >90% of the children in the age group can do the task; ◌ >75% of the children in the age group can do the task; CS: Continue Score; PEDI: Pediatric Evaluation of Disability Inventory.

◯ >90% of the children in the age group can do the task; ◌ >75% of the children in the age group can do the task; CS: Continue Score; PEDI: Pediatric Evaluation of Disability Inventory.

◯ >90% of the children in the age group can do the task; ◌ >75% of the children in the age group can do the task; CS: Continue Score; PEDI: Pediatric Evaluation of Disability Inventory.

In the mobility domain, child P8 only performed inadequately in bus transfers (2 of 5 items), shower transfers (1 of 5 items), indoor locomotion (1 of 13 items), outdoor locomotion (1 of 12 items) and stair climbing (2 of 10 items). Child P10 showed inadequate performance in all mobility sets: transfers in bathroom/ chair (5 of 10 items), in car (1 of 5 item), in shower (4 of 5 items), in bed (1 of 5 item), indoor locomotion (4 of 13 items), outdoor locomotion (3 of 12 items) and stair climbing (6 of 10 items). In the social function domain, this child had limitations in the following items: functional communication use (1 of 5 items), expressive communication complexity (2 of 5 items), interactive social games playing with adults (2 of 5 items), playing with objects (4 of 5 items), self-information (2 of 10 items), housework (1 of 5 items), self-protection (2 of 5 items), and community function (1 of 5 items).

There was no correlation between scores in self-care and mobility domains with any of the analyzed independent variables (Table 3). The social function domain score was the only one that showed a significant correlation: the score was higher (better) for children who had a unilateral injury (p = 0.05).

**Table 3.** PEDI functional domains scores related to patients’ characteristics (categorical)*.

Patients characteristics (n=14)		SCFS score	P-value	MOFS score	P-value	SFFS score	P-value
Age at stroke	< 1 year	37.8 ± 8.9	0.37	36.8 ± 5.7	0.90	53 ± 3.6	0.14
	> 1 year	37.4 ± 19.5		35.9 ± 20.2		45.5 ± 13	
Sex	Feminine	43.1 ± 11.7	0.37	41.4 ± 10.6	0.20	50.8 ± 7.6	0.56
	Male	30.3 ± 17.5		29.5 ± 18.7		45.9 ± 14.9	
Injury site	Deep	29.3 ± 14.9	0.26	25.8 ± 15.3	0.10	47.6 ± 16.2	0.79
	Lobar/Infratentorial	42.2 ± 14.2		42.1 ± 12.4		49.3 ± 8.2	
Stroke type	Ischemic	34 ± 13.5	0.24	30.9 ± 13.7	0.20	48.3 ± 13.6	0.79
	Hemorrhagic	42.4 ± 17.5		43.5 ± 15.3		49.3 ± 7.9	
Affected side	Unilateral	36.4 ± 13.3	0.37	33.6 ± 14.7	0.48	51.2 ± 12.3	0.05**
	Bilateral	40.6 ± 21.5		43 ± 16.7		42.4 ± 2.7	
Intraventricular hemorrhage	No	39.5 ± 14.1	0.27	37.1 ± 16	0.58	49 ± 11.5	0.72
	Yes	26 ± 23.3		31.1 ± 12		47.2 ± 11.8	
Hydrocephalus	No	39.5 ± 14.1	0.27	37.1 ± 16	0.58	49 ± 11.5	0.72
	Yes	26 ± 23.3		31.1 ± 12		47.2 ± 11.8	
Socioeconomic status	B	22 ± 17.8	0.10	31.8 ± 13	0.72	51.1 ± 17.2	1.00
	C	40.2 ± 14		37 ± 16		48.3 ± 10.8	

SCFS: Self-Care Functional Skills; MOFS: Mobility Functional Skills; SFFS: Social Function Functional Skills; I: Ischemic; H: Hemorrhagic; *Mann-Whitney non-parametric test; **Statistically significant.

When analyzing the PEDI's domain scores concerning hospital stay and current age, the social function domain score showed a strong negative correlation with hospital stay (r = -0.79; p = 0.001), as shown in Table 4.

**Table 4.** PEDI functional domain scores related to patients’ characteristics*.

Patients characteristics	SCFS	p-value	MOFS	p-value	SFFS	p-value
Age at assessment (years)	-0.34	0.91	-0.18	0.54	0.08	0.8
Hospital stay (days)	-0.12	0.7	-0.84	0.78	-0.79**	0.001**

SCFS: Self-Care Functional Skills; MOFS: Mobility Functional Skills; SFFS: Social Function Functional Skills; I: Ischemic; H: Hemorrhagic; *:Spearman's correlation coefficient; **: Statistically significant.

## DISCUSSION

We analyzed the functional outcome of 14 children diagnosed with stroke in early childhood. Overall, the children showed age-appropriate functional outcomes measured by the PEDI's functional domains: over 70% of them in self-care and mobility and 90% in social function. Children with bilateral injuries and longer hospital stays showed the worst scores in ​​PEDI's social function domains.

Of the 4 children with age-inappropriate self-care performance (P4, P8, P10, P14), two (P4, P8) had typical hemiparetic sequelae limitations. Child P10 showed limitations related to neuromotor and cognitive demands on self-care tasks; this is the only child with age-inappropriate social function. The child had multiple lacunar infarctions and microangiopathy due to hemolytic-uremic syndrome (HUS) during hospital stay. Previous case reports of post-HUS children aged 15 and 21 months (the same age as the child in our study) also described neuromotor and language impairments^21^. On the other hand, child P14 had no evident motor deficit; most of its limitations in self-care were related to routine care of the hemodialysis catheter due to chronic kidney disease. 

Among the children with age-inappropriate mobility (P1, P8, P10, P14), children P8 and P10 had neuromotor limitations to a lesser or greater degree. However, the low scores of children P1 and P14 may be due to a limitation on the normative scale. At the time of data collection, both children were between 5 years and 5 years and 5 months old. Within this age range, raw scores of P1 and P14 reflect an age-inappropriate performance when compared to normative scores (below 30). However, similar scores in the following age range (5 years and 6 months to 5 years and 11 months) reflected an age-appropriate performance, with normative scores greater than 30. Considering that certain scores are considered age-inappropriate in a certain age range, but identical scores are considered age-appropriate in an older age range, it is possible that the normative scores are a limitation and may require further investigation.

To the best of our knowledge, only Galvin et al.^9^ used the PEDI to assess functional outcomes after childhood stroke. However, the authors only presented each domain's average score (not the children's frequency of appropriate outcomes), precluding the comparison with our results. The children in our study presented age-appropriate average scores in self-care, mobility, and social function domains, although scores were close to the lower limit of normality. This is in accordance with previous childhood stroke studies showing good mobility, even with hemiparesis and requiring orthoses^13^^,^^22^. Cooper et al.^23^ found good motor recovery (fine and gross motor function) in children (0-19 years) over the first year after stroke, with more pronounced improvement in preschool-age children^23^.

Conversely, Galvin et al.^9^ observed that children with ischemic stroke showed lower levels of functional skills in all domains: self-care (70.36 ± 30.82), mobility (77.97 ± 27.58) and social function (74.88 ± 30.57). Previous studies have demonstrated that children with stroke showed unsatisfactory performance for ADLs, communication, and social function activities^12^^,^^22^, especially in early-age stroke children^22^, which does not corroborate with our results. However, the age range of our sample may explain such discrepancy. Specifically, the studies mentioned above involved children up to 16 ^9^ and 18 years of age^12^^,^^22^, while our study included children up to 7.5 years of age. In this age range, deficits in complex communication, ADLs, and social skills may not be as obvious. Also, the development of each of these skills influences that of the other; Cooper et al.^23^ described that communication skills may influence ADL throughout children's development^23^. 

Similarly, studies suggest that children present reduced communication skills, as well as the cognition-related and social functions after stroke. Friefeld et al.^24^ observed that the quality of life (QoL) related to physical aspects and the domestic environment was less impaired and the QoL related to school and playing were more affected, mainly due to cognitive and behavioral elements^24^. Based on the literature, about half of the children with childhood stroke present limitations on school activities and participation and require specialized education^12^^,^^22^^,^^25^^,^^26^. Additionally, children present significant impairment on cognition-related functions, intelligence, memory, language, and social function^6^^,^^26^^,^^27^. Studies have also shown that cognitive social and task performances were worse in children who had stroke at a younger age^6^^,^^26^^,^^28^.

The significant negative correlation between length hospital stay and social function may be reflecting the impact of the chronic underlying condition on socialization. Of the four children with longer hospital stays, only one had evident neuromotor sequelae (P10). However, all of them had a systemic condition (chronic liver or renal disease/ vasculopathy with toes amputation) with potential clinical complications, which could affect the dynamics of family functioning related to child care. Supporting this speculation, Christerson and Strömberg^12^ reported that post-stroke children's outcomes were more dependent on etiology and recurrences (rebleeding, metabolic diseases, Moya-Moya syndrome) than the age at stroke or injury site. A weak social competence may not be due solely to brain injuries but to the child's experience with the particularities of the illness in their social world^29^. 

 The relationship between worse scores in social function and bilateral injury may be related to the children's interhemispheric neuroplasticity process. Mosch et al.^30^ observed that, differently from adults, children with right cerebral hemisphere (RCH) injury did not present reduced social function, suggesting a positive plasticity process in children. However, children with left cerebral hemisphere (LCH) injury presented worse social function (and better language function) than adults with an LCH injury. Authors speculate that after LCH injury in children, the plasticity process may involve recruiting RCH contralateral areas previously intended for social function to preserve LCH functions, such as language^30^. In our study, social function skills did not correlate with LCH injury. This may be due to the younger age of our sample. The complexity of preschool social demands is low and usually related to lower-order skills, which can be attributed to less complex neural networks and often have good functional recovery^31^^-^^33^. The more complex skills that are usually impaired after childhood stroke (executive, cognitive, social skills) are needed as children grow up and move into more socially complex environments like school, college, and work^6^^,^^22^^,^^24^^,^^26^^,^^34^. 

The age-appropriate functional outcomes in childhood after stroke found in our study should be interpreted with caution and considered especially from a functional perspective. The finding shows that preschool children can functionally keep up with their peers despite stroke. The PEDI score is influenced by the ICF model, in which the functionality in a specific domain results from the interaction between health condition and the contextual factors (environmental and personal)^4^. Functional performance in a given task is influenced not only by the child's characteristics but also by the task's specific demands and the environmental aspects with which the child interacts^35^. According to the ICF, effective rehabilitation requires going beyond pathological conditions/sequels and promoting the individual's activity and participation^36^.

One limitation of our study is the small number of participants. On the other hand, this allowed rich inferences from the occasional analysis of isolated cases. As an implication for research, our study reinforces the importance of the interhemispheric neuroplasticity process in children and the impact of the chronic nature of the underlying condition/stroke on social functions. Moreover, it has implications for clinical practice by supporting recovery based on activity/participation levels and increase surveillance of children with stroke, mainly related to social function, even when there are no obvious deficits at discharge.

In summary, preschool children showed age-appropriate functional outcomes on self-care, mobility, and social function domains after stroke. However, children with bilateral injuries and longer hospital stays had the worst scores in social function domains. We recommend focusing on functional rehabilitation to promote activities and participation and to monitor the development of the children's social skills after stroke.
